# Adults with Prader–Willi syndrome exhibit a unique microbiota profile

**DOI:** 10.1186/s13104-021-05470-6

**Published:** 2021-02-06

**Authors:** Wendy J. Dahl, Jérémie Auger, Zainab Alyousif, Jennifer L. Miller, Thomas A. Tompkins

**Affiliations:** 1 Department of Food Science and Human Nutrition, University of Florida, 359 Newell Drive, Gainesville, FL 32611 USA; 2 Rosell Institute for Microbiome and Probiotics, 6100 Royalmount, Montreal, QC H4P 2R2 Canada; 3 Division of Endocrinology, Department of Pediatrics, College of Medicine, University of Florida, 1600 SW Archer Road, Gainesville, FL 32610 USA

**Keywords:** Prader–Willi syndrome, RF39, *Blautia*, Tenericutes, Microbiota, 16S rRNA

## Abstract

**Objective:**

Adults with Prader–Willi syndrome (PWS) require less energy intake to maintain body weight than the general adult population. This, combined with their altered gastrointestinal transit time, may impact microbiota composition. The aim of the study was to determine if the fecal microbiota composition of adults with PWS differed from non-affected adults. Using usual diet/non-interventional samples, fecal microbiota composition was analyzed using 16S rRNA gene amplicon sequencing and data from adults with PWS were merged with four other adult cohorts that differed by geographical location and age. QIIME 2™ sample-classifier, machine learning algorithms were used to cross-train the samples and predict from which dataset the taxonomic profiles belong. Taxa that most distinguished between all datasets were extracted and a visual inspection of the R library PiratePlots was performed to select the taxa that differed in abundance specific to PWS.

**Results:**

Fecal microbiota composition of adults with PWS showed low *Blautia* and enhanced RF39 (phyla Tenericutes), Ruminococcaceae, *Alistipes*, Erysipelotrichacaea, *Parabacteriodes* and *Odoribacter.* Higher abundance of Tenericutes, in particular, may be a signature characteristic of the PWS microbiota although its relationship, if any, to metabolic health is not yet known.

## Introduction

Prader–Willi syndrome (PWS) is a genetic disorder characterized by lower energy requirement, lack of satiety, and hyperphagia, which together lead to obesity if food intake is not strictly controlled [1]. Although reported total fiber intake of adults with PWS [2, 3] is similar to the general population [4], constipation is common in this patient population [5]. Microbiota composition may be altered by constipation; the evidence suggests decreased *Bifidobacterium* [6, 7], *Lactobacillus* [7] and *Bacteroides* [6] compared to healthy controls. Further, individuals with constipation-predominant irritable bowel syndrome (IBS) are also reported to have lower *Bifidobacterium* and *Lactobacillus* spp., as well as lower *Roseburia*–*E. rectale* taxa and higher sulfate-reducers [8]. However, in women, fecal microbiota profile was not associated with constipation, but to colonic transit time [9]. Adults with PWS display delayed mean intestinal transit time compared to healthy controls [5], suggesting motility issues which may impact microbiota profile.

In adults with PWS, the microbiota composition has been shown to differ from controls matched for age, gender, and body fat mass index. Specifically, those with PWS had a higher abundance of *Akkermansia*, *Desulfovibrio* and taxa of Tenericutes and Archaea, but a lower abundance of *Dorea* [10]. However, the microbiota composition of these subjects with PWS did not differ significantly from that of their parents. It remains unclear as to whether the microbiota composition of individuals with PWS is characteristic of the syndrome or the environment. Given the interconnections between microbiota, its metabolism and metabolic health, insight into the microbiota profile of PWS, considered a model of hyperphagia [11], is of interest. The aim of this exploratory analysis was to determine if the microbiota composition of adults with PWS differed from unaffected adults, independent of geographical location and age.

## Main text

### Methods

De-identified 16S profiles from usual diet/non-interventional fecal samples of 25 adults (34.9 ± 10.2 years; 60% female) with genetically confirmed PWS and residing in Florida [3, 12] were compared to those of healthy adults residing in Canada (n = 151; 35.2 ± 10.1 years; 61% female) [13], adults with IBS residing in Canada (n = 263; 41.8 ± 15.2; 79% female) [14], healthy young adults residing in Florida, USA (n = 68; 23.2 ± 3.5; 63% female) [15], and healthy older women residing in Florida (n = 26; 73.7 ± 5.6 years) [16]. All source data originated from the same lab environment and were subsequently treated with the same bioinformatics processing. Details of DNA extraction [3, 16] and methods for community-wide taxonomic profiling via 16S amplicon sequencing of the fecal samples, carried out in the source studies, were previously reported [3], i.e. no DNA extraction or sequencing was conducted for the purposes of the present analysis. The Institutional Review Board of the University of Florida approved this analysis of de-identified 16S profiles as exempt. The data in fastq format were imported into one QIIME artefact (demux.qza) [17]. All reads were quality filtered with same parameters and trimmed at 240 bp on the forward read. Using the Deblur denoiser (implemented as a QIIME 2 module), the amplicon sequence variant (ASV) abundance tables and representative sequences were generated. The representative sequences were merged to make the taxonomic profiles using the ‘taxonomy.qza’ trained on the GreenGenes database. The ASVs from the abundance table were attributed to known taxonomic names and compiled at the genus level for further analyses.

Using QIIME’s visualization tools, the principal coordinates analysis (PCoA), weighted UniFrac, and individual taxonomic profiles were generated and examined [17–36]. Group differences were highlighted using QIIME 2 sample-classifier. This module allowed machine learning algorithms to cross-train on the samples and predict the label (here the label is ‘from which dataset does the taxonomic profile comes from’). The important features used by the algorithm (those taxa that distinguish the most between dataset) were extracted and the taxa list was used for further exploration. Each important feature was plotted using the R library PiratePlot (R version 3.5.3). Since this taxa list was not made to single out PWS, but rather distinguish between all datasets, a visual inspection of the plots was performed to select the taxa that differed in abundance specific to PWS. Eight PWS-specific taxa appeared in the top 36 important features.

### Results

**Fig. 1:**
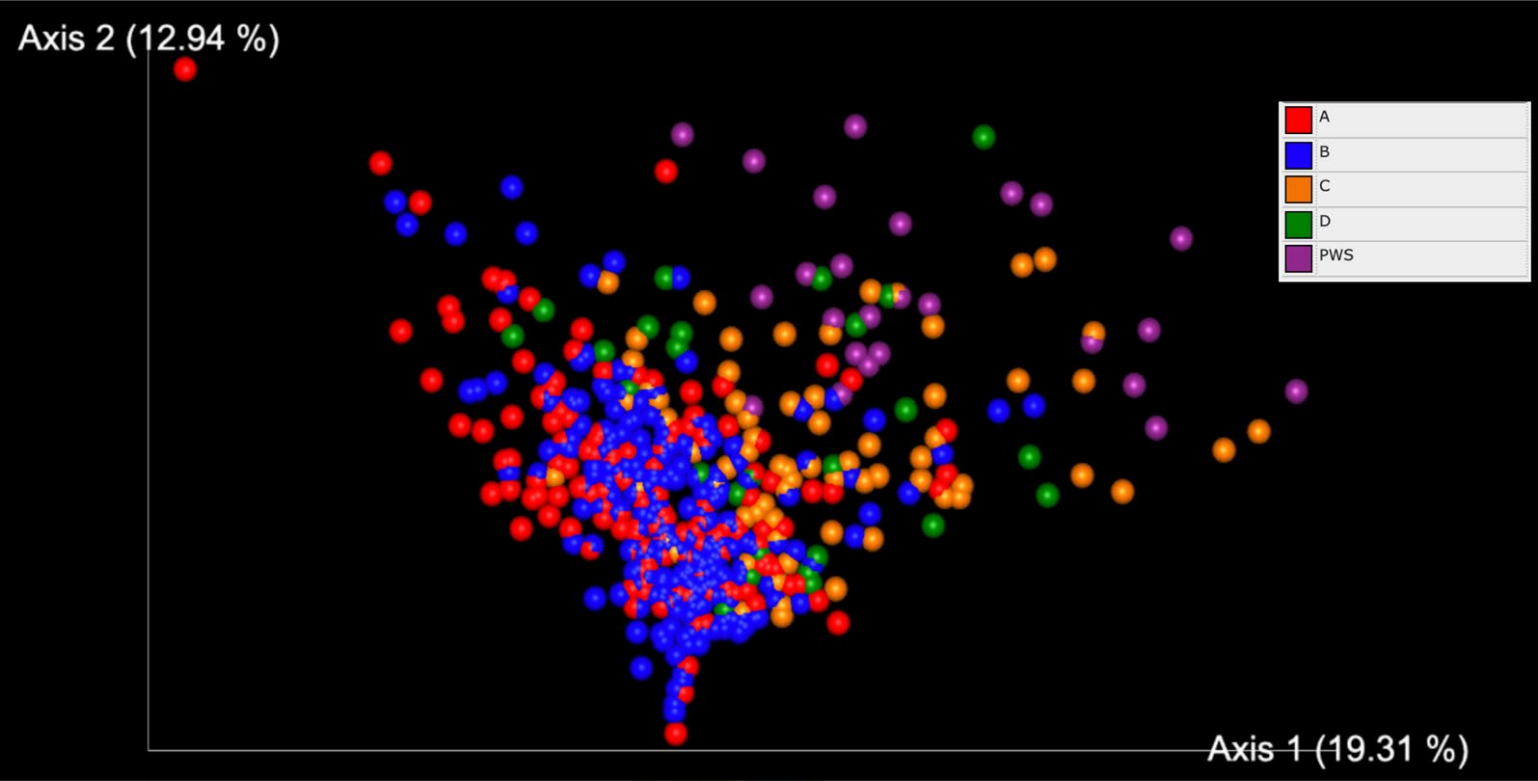
Beta diversity by Weighted UniFrac Principal Coordinates Analysis (PCoA) of the fecal microbiome data sets of adults with Prader–Willi syndrome (PWS) and (A) adults residing in Canada; (B) adults with irritable bowel syndrome residing in Canada, (C) adults residing in Florida, USA, (D) older women residing in Florida

**Fig. 2:**
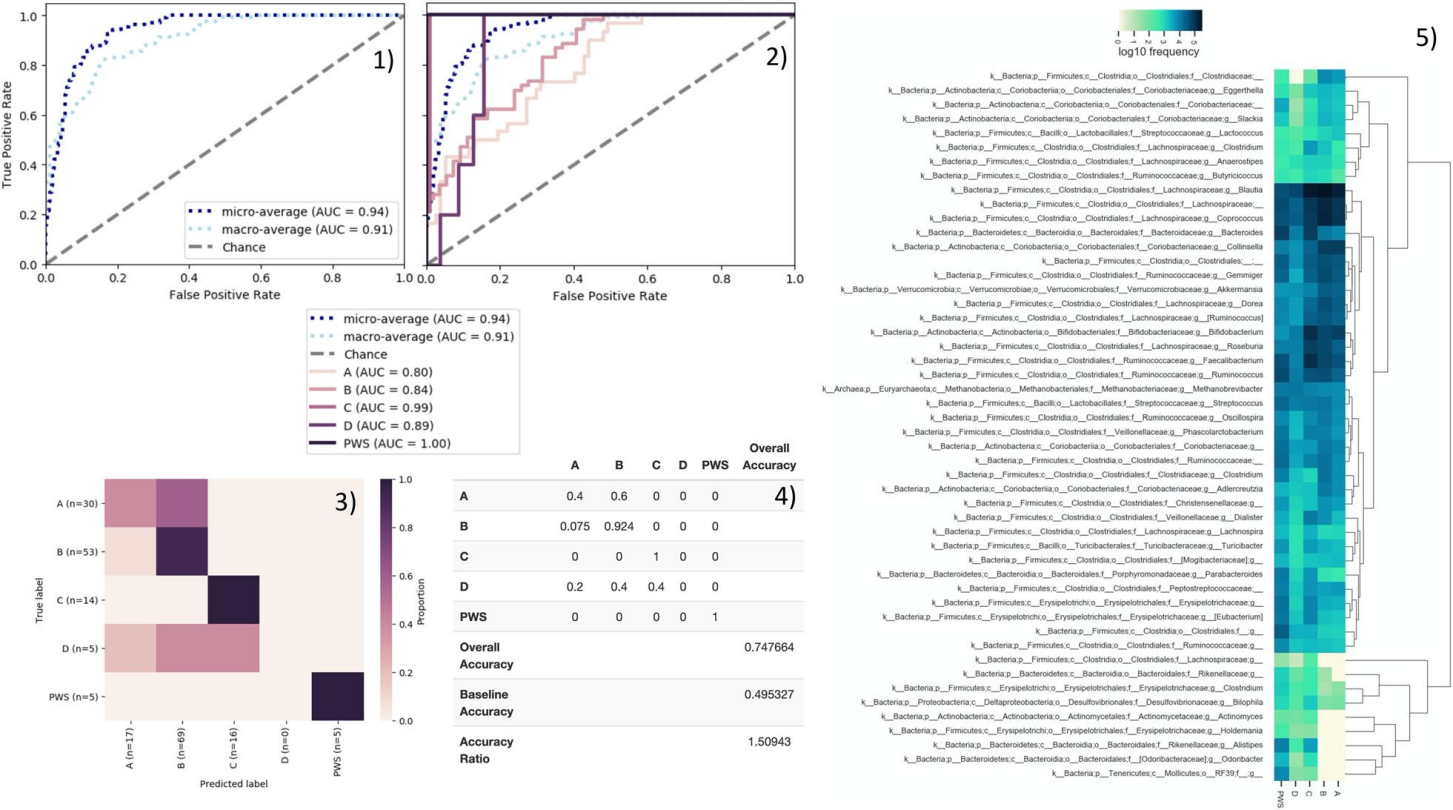
Receiver Operating Characteristic (ROC) curves measuring the performance of the Machine Learning classification model at all classification thresholds of **1** average scores and **2** per-class. Model optimization curves are the true positive rates over the range of false positive rates. **3** Model accuracy and **4** overall and baseline accuracy for the Extra-Tree classification of the merged dataset samples for adults with Prader–Willi syndrome (PWS) and adult cohorts (A) residing in Canada, (B) with irritable bowel syndrome residing in Canada, (C) residing in Florida, USA, and (D) older women residing in Florida. **5** Heatmap visualization depicting the relative abundances from fecal samples of the important taxa used for the classification of adults with Prader–Willi syndrome versus non-affected adult groups

The Weighted UniFrac PCoA from QIIME of the merged datasets was visualized using Emperor and color coded according to the sample’s clinical trial provenance and is shown in Fig. 1. Each data point represents a fecal sample and the distance matrix between them based on ASV counts and phylogeny. The PWS samples seem to cluster (upper right portion) and the other samples all show significant overlap indicates that the PWS profiles are different from those of the other adults. Conversely, PWS profiles have similarities not shared by the other groups. On this basis, a machine learning algorithm was implemented to classify the profiles on sample origin and highlight the most explicative taxa. Figure 2 shows the Machine Learning model—accuracy results for the Extra-Tree classification trained on the merged dataset samples taken at baseline. The confusion matrix shows strong accuracy scores on the main diagonal and a good overall accuracy result of 74.8% and an accuracy of 100% for the PWS predictions alone. The training was done on an 80–20% training-to-testing cross validation ratio. QIIME’s sample classifier module heatmap visualization is shown in Fig. 2, depicting the relative abundances of the important taxa used for the classification for each of the groups compared.

**Fig. 3:**
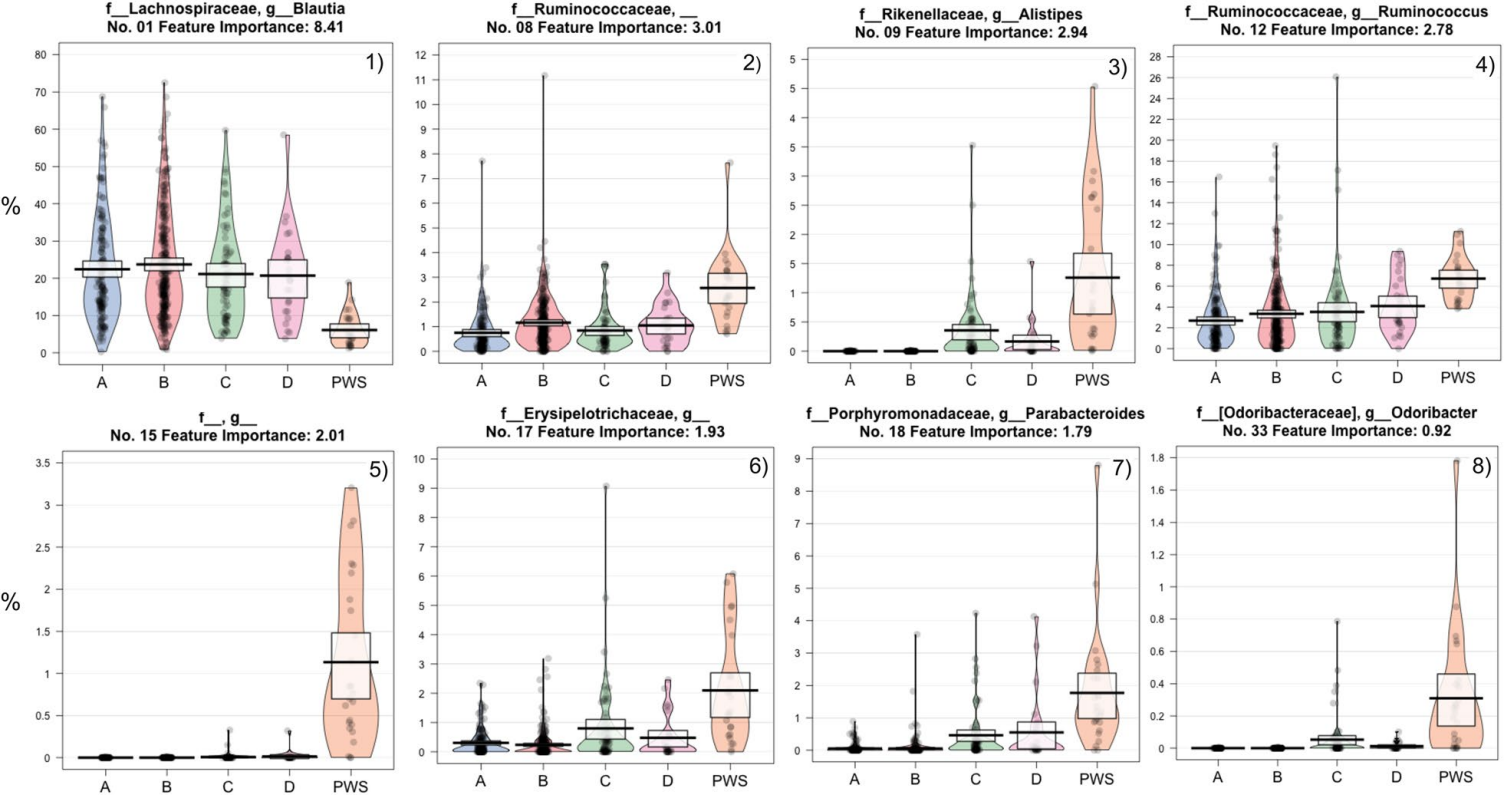
PiratePlots of the relative abundances of 8 taxa, specifically **1** genus *Blautia*, **2** family Ruminococcaceae, **3** genus *Alistipes* **4** genus *Ruminococcus*, **5** order RF39 **6** family Erysipelotrichaceae **7** genus *Parabacteroides*, and **8** genus *Oridobacter,* which by manual, visual inspection, represent apparent differences between the Prader–Willi syndrome (PWS) microbiota profiles compared to adults (A) residing in Canada, (B) adults with irritable bowel syndrome residing in Canada, (C) adults residing in Florida, USA, and (D) older women residing in Florida

The list of important taxa (and associated importance score) was obtained from the ‘feature_importance.qza’ file generated by the classifier and used for individual taxa plotted in Additional file 1: Fig. S1. Note that the model optimized for overall performance and thus, many of the 36 important features taxa shown are used by the model to distinguish between non-PWS samples. The selection of PWS explaining taxa by manual visualization (taxa graphs where the PWS is obviously different from all the others) suggests 8 taxa of the 36 important features are linked with PWS microbiota profiles (Fig. 3). From the 8 PWS-specific taxa, only the genus *Blautia* (ranked 1) was lower in PWS than other cohorts. This is of interest given *Blautia* was the most abundant genus in all datasets, averaging 21% of the total sample composition. Ruminococcaceae appeared twice (rank 8 and 12) at different taxonomic levels. The genus *Alistipes* (family Rikenellaceae, phylum Bacteroidetes) ranked 9 and is clearly higher in PWS and nearly absent in some others. Erysipelotrichacaea, *Parabacteroides* (family Porphyromonadaceae) and *Odoribacter*, ranked 17, 18 and 33, respectively, also appeared higher in the PWS dataset. Finally, rank 15 was the order RF39 (phylum Tenericutes, class Mollicutes) and it seems to be strongly linked with PWS, as the maximum values of relative abundances for this taxon in samples of non-affected adult cohorts are much lower than those of PWS. Of note, rank 3 (Additional file 1: Fig. S1) suggests *Bifidobacterium* abundance of the PWS profiles were similar to the older women but lower than the other adult cohorts.

### Discussion

Previous research suggests that adults with PWS may harbor a microbiota composition with unique characteristics [10]. In the present study, the abundance of *Bifidobacterium* was lower in PWS, with the exception of the older women who exhibited similarly low levels. This finding was not unexpected given that suppression of *Bifidobacterium* has been reported in individuals with constipation [7] and some of the adults with PWS reported slow transit stool form, suggestive of constipation [3]. This finding is concerning though given the protective role of *Bifidobacterium* spp. in maintaining intestinal eubiosis and barrier function [37]. PWS adults exhibited higher abundance of Tenericutes (order RF39), *Alistipes*, *Parabacteroides*, and *Odoribacter*, as well as Ruminococcaceae and Erysipelotrichaceae, compared to the non-affected adults. Of interest, RF39 was one of the taxa identified by Olssen et al. in PWS adults [10] and by Peng et al. in children with PWS compared to matched controls [38]. The relevance of higher levels of Tenericutes in PWS adults is not known; however, the abundance of *Alistipes* and *Parabacteroides* have been negatively associated with cardiometabolic indices such as serum lipids, blood glucose, and blood pressure [39]. Additionally, Ruminococcaceae has been negatively associated with metabolic syndrome [40] and lower long-term weight gain [41]. Conversely, abundance of Erysipelotrichaceae has been associated with obesity and lipid metabolism, and specific taxa within this family may be inflammatory and immunogenic [42]. Both *Odoribacter* and *Alistipes* have been associated with diet quality [43]. Olsson et al. showed that adults with PWS had lower abundance of *Dorea* compared to obese controls [10]. Similarly, Peng et al., by random forest analyses, also identified a difference in *Dorea* in children with PWS when compared to controls [38]. *Dorea* was not an identifying taxon for PWS in the present study. However, the adults with PWS profiled in this analysis had much lower body mass index (BMI) [3] than the subjects in the Olsson study. Lower abundance of *Blautia* in adults with PWS presents as an interesting enigma. Three genera in the Lachnospiraceae family have been shown to be positively correlated with BMI, namely *Blautia*, *Dorea*, and *Ruminococcus* [44]. *Blautia* abundance has been shown to be inversely associated with visceral fat area—after adjustment for age, BMI, and other lifestyle-related factors [45]. Adults with PWS present with lower visceral fat area compared to healthy adults [46], which is thought to contribute to their reduced risk of developing type 2 diabetes [47]. Thus, given their weight status and typically lower visceral fat, it might be expected that individuals with PWS would have higher abundance of *Blautia*. Of note, *Blautia* abundance also has been correlated with higher serum insulin and impaired lipid metabolism [48], suggesting a benefit for low abundance. The finding of lower levels of *Blautia* in adults with PWS may, therefore, correlate with their relative insulin sensitivity despite frequent obesity. In summary, the microbiota profile findings may suggest benefit related to the reported cardiometabolic protection in PWS [47].

*Blautia* spp*.* utilize dietary carbohydrates [49], thus the restricted carbohydrate intake of the PWS adults [3] may have contributed to lower *Blautia* abundance. Lower abundance of *Blautia* has been shown in athletes consuming a higher protein, lower carbohydrate diet, compared to sedentary controls [50]. Although the absolute intake of protein, fat and carbohydrate of the adults with PWS is a fraction of the intake of athletes, the percentage of energy from protein was similar [3], and thus macronutrient composition may contribute to *Blautia* abundance. However, no association between protein, fat, carbohydrate or fiber intake with *Blautia* abundance was found in a large cross-sectional study [45]. Of further interest is the relationship between *Blautia* abundance and gastrointestinal symptoms. IBS patients, whose gastrointestinal symptoms decreased with a low-FODMAP diet, had higher abundance of *Blautia* [51], suggesting visceral sensitivity in these individuals. In contrast, individuals with PWS exhibit a high tolerance to pain and discomfort [52]. The adults with PWS who provided fecal samples for this analysis reported minimal gastrointestinal discomfort [3], similar to healthy individuals [15, 53]. The possibility of a relationship between *Blautia* abundance and visceral sensitivity requires further investigation.

The results of this analysis provide further evidence that the microbiota composition of individuals with PWS differs from that of unaffected individuals, notably with the presence of higher Tenericutes, specifically the order RF39, although the implications to health are unknown. Further, it may be interesting to explore the relationship between *Blautia* abundance and visceral sensitivity, as well as metabolic health, in PWS and other patient populations. Given their low prevalence of *Bifidobacterium* spp., the PWS population may benefit from synbiotic supplementation.

## Limitations

This study had limitations. Foremost, the study was undertaken as a post hoc analysis. Its major limitation was the use of a merged dataset to undertake the comparison of PWS to non-affected adults, as the fecal samples were not processed in the same batch. However, all samples were all processed using exactly the same methodology in terms of collection, storage, sequencing, and bioinformatics. A single database, Greengenes, was used; results may differ if data was analyzed using another database such as SILVA.

## Supplementary Information

**Additional file 1: Figure S1.** PiratePlots of all taxa distinguishing groups of the merged datasets including: (A) adults residing in Canada; (B) adults with irritable bowel syndrome residing in Canada, (C) adults residing in Florida, USA D) older women residing in Florida, and PWS) adults with Prader–Willi syndrome.

## Data Availability

The dataset of the PWS adults is available at: https://www.ncbi.nlm.nih.gov/bioproject/?term=PRJNA669563. The full dataset generated and analyzed during the current study is not publicly available but deidentified data are available from the corresponding author upon reasonable request.

## Abbreviations

BMI: Body mass index
IBS: Irritable bowel syndrome
PCoA: Principal coordinates analysis
PWS: Prader–Willi syndrome
