# Supplementary figures and images for: Adults with Prader–Willi syndrome exhibit a unique microbiota profile
Source: BMC Res Notes. 2021 Feb 6;14:51. doi: 10.1186/s13104-021-05470-6 (PMC7866703; doi:10.1186/s13104-021-05470-6)

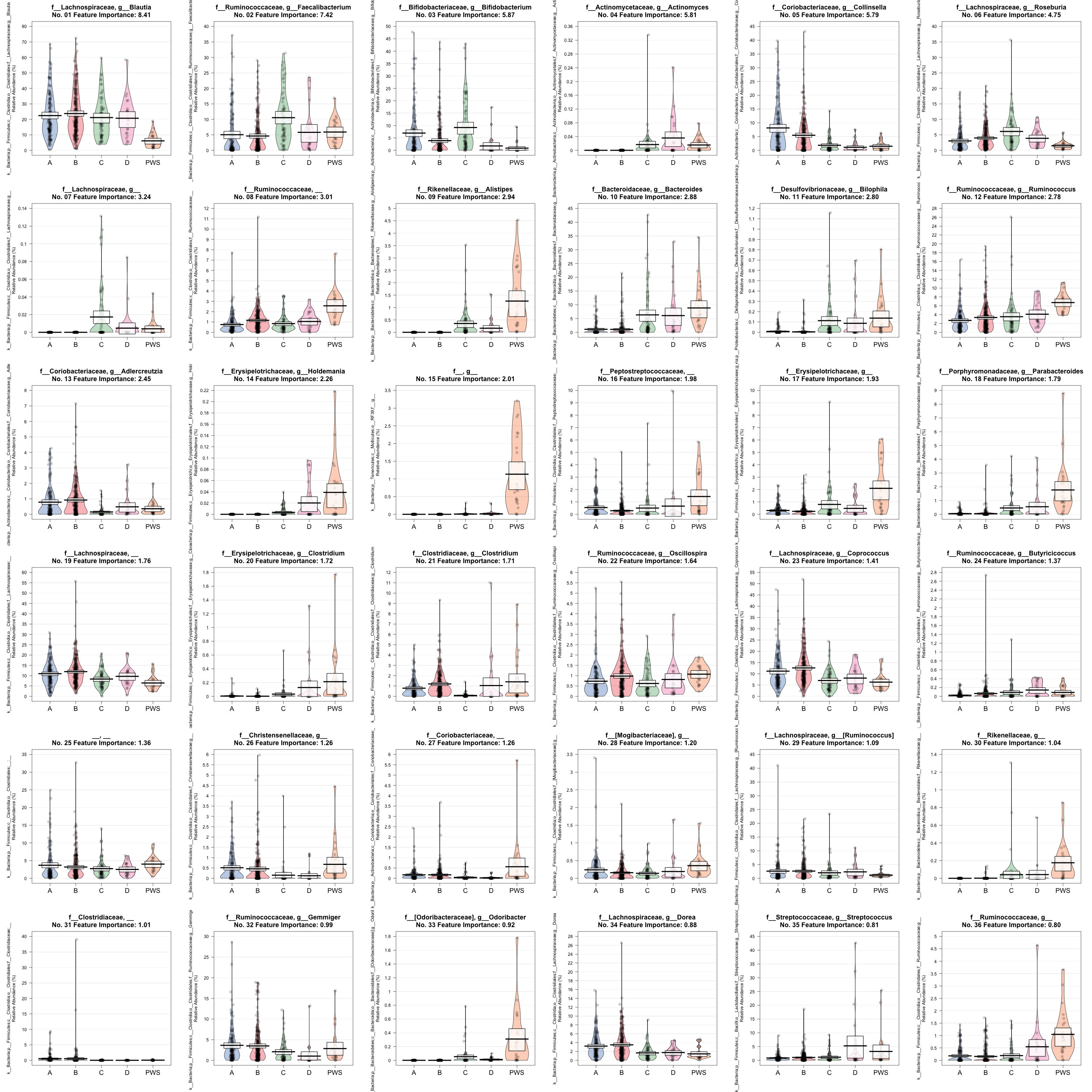

Supplement: Supplementary file 1 — Additional file 1: Figure S1. PiratePlots of all taxa distinguishing groups of the merged datasets including: (A) adults residing in Canada; (B) adults with irritable bowel syndrome residing in Canada, (C) adults residing in Florida, USA D) older women residing in Florida, and PWS) adults with Prader–Willi syndrome. [file 13104_2021_5470_MOESM1_ESM.png]
